# Vertigo Perception and Quality of Life in Patients after Surgical Treatment of Vestibular Schwannoma with Pretreatment Prehabituation by Chemical Vestibular Ablation

**DOI:** 10.1155/2016/6767216

**Published:** 2016-12-08

**Authors:** Zdeněk Čada, Zuzana Balatková, Martin Chovanec, Ondřej Čakrt, Silvie Hrubá, Jaroslav Jeřábek, Eduard Zvěřina, Oliver Profant, Zdeněk Fík, Martin Komarc, Jan Betka, Jan Kluh, Rudolf Černý

**Affiliations:** ^1^Department of Otorhinolaryngology and Head and Neck Surgery, 1st Faculty of Medicine, Charles University and Motol University Hospital, Postgraduate Medical School, Prague, Czech Republic; ^2^Department of Otorhinolaryngology, 3rd Faculty of Medicine, University Hospital Kralovske Vinohrady, Charles University, Prague, Czech Republic; ^3^Department of Rehabilitation and Sports Medicine, 2nd Faculty of Medicine, Charles University and Motol University Hospital, Postgraduate Medical School, Prague, Czech Republic; ^4^Department of Neurology, 2nd Faculty of Medicine, Charles University and Motol University Hospital, Postgraduate Medical School, Prague, Czech Republic; ^5^Department of Anthropometrics and Methodology, Faculty of Physical Education and Sport, Charles University, Prague, Czech Republic

## Abstract

Surgical removal of vestibular schwannoma causes acute vestibular symptoms, including postoperative vertigo and oscillopsia due to nystagmus. In general, the dominant symptom postoperatively is vertigo. Preoperative chemical vestibular ablation can reduce vestibular symptoms postoperatively. We used 1.0 mL of 40 mg/mL nonbuffered gentamicin in three intratympanic installations over 2 days, 2 months preoperatively in 10 patients. Reduction of vestibular function was measured by the head impulse test and the caloric test. Reduction of vestibular function was found in all gentamicin patient groups. After gentamicin vestibular ablation, patients underwent home vestibular exercising for two months. The control group consisted of 10 patients who underwent only home vestibular training two months preoperatively. Postoperative rates of recovery and vertigo in both groups were evaluated with the Glasgow Benefit Inventory (GBI), the Glasgow Health Status Inventory (GHSI), and the Dizziness Handicap Inventory questionnaires, as well as survey of visual symptoms by specific questionnaire developed by us. There were no statistically significant differences between both groups with regard to the results of questionnaires. Patients who received preoperative gentamicin were more resilient to optokinetic and optic flow stimulation (*p* < 0.05). This trial is registered with clinical study registration number NCT02963896.

## 1. Introduction

In most cases, surgery for vestibular schwannoma leads to acute vestibular dysfunction in postoperative period [1–4]. The dominant symptoms are vertigo, nausea, and postural imbalance. After each acute vestibular asymmetry, central compensation and recalibration commence and usually subside over weeks to months. Vestibular rehabilitation plays a key role in the process of compensation [5, 6]. Vestibular rehabilitation reduces spontaneous nystagmus and improves posture. Rehabilitation programs consist of eye movement and postural exercises. Age, internal, psychiatric, and neurologic comorbidities slow down the process of compensation. One promising possibility to speed up postoperative vestibular rehabilitation is vestibular prehabituation by chemical peripheral vestibular ablation with ototoxic gentamicin [7]. The presumption of prehabituation is decreasing labyrinth function before surgery and allowing for faster compensation in postoperative time. In this report, we present our experience of chemical vestibular prehabituation, as evaluated by questionnaires. The validated Glasgow Benefit Inventory (GBI) [8] measures change in health status after otorhinolaryngological interventions. The Glasgow Health Status Inventory (GHSI) [8] is validated questionnaire that measures the effect of a health problem on quality of life and allows for cross-comparison among many health conditions and different health interventions. The GBI is sensitive to change in health status brought about by a specific event (e.g., an operation) while the GHSI provides a general measure of health status. The Dizziness Handicap Inventory (DHI) [9] is used to assess the impact of dizziness on quality of life.

## 2. Methods

In this study, 20 patients (10 male, 10 female, mean age 50, range 33–65 years) who underwent surgery for vestibular schwannoma (retrosigmoid approach) between 2014 and 2015 were included. All patients were preoperatively examined and found to be indicated for surgery. All patients had profound hearing loss on the tumor side (pure tone average >60 dB). A caloric test was performed in all patients prior to surgery and canal paresis was measured. The size of the tumor was classified according to the Koos classification (Table 1). Patients were randomly divided into either a control group (10 patients, 4 female, 6 male) or the gentamicin group (10 patients, 6 female, 4 male). 40% of control subjects and 70% of ITG subjects had canal paresis >25%. All patients had negative head impulse test. Patients in the gentamicin group received (at least two months before surgery) three transtympanic injections of 0.5 mL of nonbuffered gentamicin (40 mg/mL), with each instillation given over 48 hours.

After 3 to 4 weeks, patients were evaluated by both the head impulse test and the caloric test to ensure that vestibular ablation was achieved. In case that vestibular ablation was not reached next doses of gentamicin were injected. Both groups began to practice vestibular home exercises two months before surgery. Patients were instructed to practice the home based vestibular training program adopted for patients with acute vestibular loss. Program included gaze stability exercises, smooth pursuit and saccadic eye movements, and postural exercises to improve balance control and gait stability. This study was approved by institutional ethics committee of the University Hospital Motol and all patients provided informed consent prior to study commencement. All patients were required to complete DHI and GHSI questionnaires 3 months preoperatively (time 1), two months preoperatively after gentamicin installation and/or vestibular exercises (time 2), and 3 months postoperatively (time 3). GBI questionnaires were completed two months after gentamicin vestibular ablation (time 2) and three months postoperatively (time 3) in both groups. Questionnaires were translated into the Czech language. We designed an additional questionnaire (nine questions, (A)–(I)), based on the most frequent complaints presenting in our clinical practice. Our questionnaire included items not specifically mentioned in the other standard questionnaires used in this study. Patients completed this additional questionnaire 3 months before surgery, 3 weeks after surgery, 3 months after surgery, and one year after surgery.

*Questions (A)–(I)*

- Do you have instability with, or does faster rotational motion bother you (e.g., rotates head from side to side when crossing the road)?
- Do you have instability with, or does walking on uneven surfaces bother you (e.g., walking up the stairs/walk in the snow)?
- Do you have instability with, or does quickly changing position bother you (e.g., lying on a bed/getting up/recumbent)?
- Do you have instability with, or does walking in darkness/twilight bother you?
- Do you have instability with, or does reading while driving bother you (the ability to keep eyes when walking)?
- Do you have instability with, or does shopping in a supermarket bother you (rapid changes in products on the shelves)?
- Do you have instability with, or does a greater amount of auditory and visual sensations bother you (e.g., shopping centers)?
- Do you have instability with, or does longer reading bother you?
- Do you have instability with, or does watching TV bother you?

## 3. Questionnaires

The GBI questionnaire consists of 18 questions. The response to each question is based on a five-point Likert scale, ranging from a large deterioration to a large improvement in health status. The GBI questionnaire is scored into a total score, and also three subscales: a general subscale (12 questions), a social support subscale (three questions), and a physical health subscale (three questions). Score ranges were calculated and varied from −100 to +100. Score all questions so that a score of 1 is given to the answer with the worst change in health status and 5 to the answer with the best change in health status.

The GHSI questionnaire contains 18 questions; again, the response to each question is based on a five-point Likert scale ranging from high to low health status. It is also scored into a total score and three subscales: general, social, and physical health subscales. All these scores range from 0 to +100.

Score all questions so that a score of 1 is given to the answer with the worst change in health status and 5 to the answer with the best change in health status.

The DHI contains 25 items and the range score is from 0 to +100, with a higher score indicating a more severe handicap.

The additional questionnaire (see questions (A)–(I)) was prepared by neurotologists from the Department of Otorhinolaryngology and Head and Neck Surgery of the 1st Faculty of Medicine, Charles University in Prague, Faculty Hospital Motol, Postgraduate Medical School. Each question had a score ranging from 1 to 4, with a higher score indicating a more severe handicap. Every question was statistically compared between the gentamicin and control groups.

## 4. Statistical Analysis

Basic descriptive statistics (mean, median, confidence interval, standard deviation, interquartile range, skewness, and kurtosis) were computed for all variables, which were subsequently tested for normality using the Kolmogorov-Smirnov and Shapiro-Wilk tests. Differences in analyzed variables (age, canal paresis, length of surgery, size of tumor, GHSI total and subscale scores, GBI total and subscale scores, DHI scoring, and nine extra questions) between groups were evaluated by the independent-group *t*-test. The Mann-Whitney *U* test was used as a nonparametric alternative. Changes in outcomes measured by the above-mentioned questionnaires at different time points were assessed by two-way repeated ANOVA (time × group), followed by the LSD post hoc comparisons. A *p* value less than 0.05 was considered to be statistically significant. Statistical analyses were performed using SPSS version 23 (SPSS Inc., Chicago, IL, USA) statistical software.

## 5. Results

**Figure 1:**
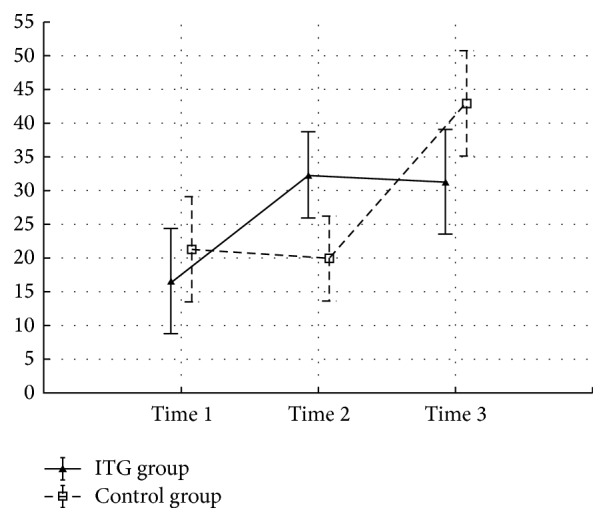
Means and SEM for DHI questionnaire. Time 1: 3 months before surgery, time 2: 2 months before surgery, after pretreatment in the ITG group, and time 3: 3 months after surgery. ANOVA 2-way (time × group) interaction effect *p* value < 0.05.

**Figure 2:**
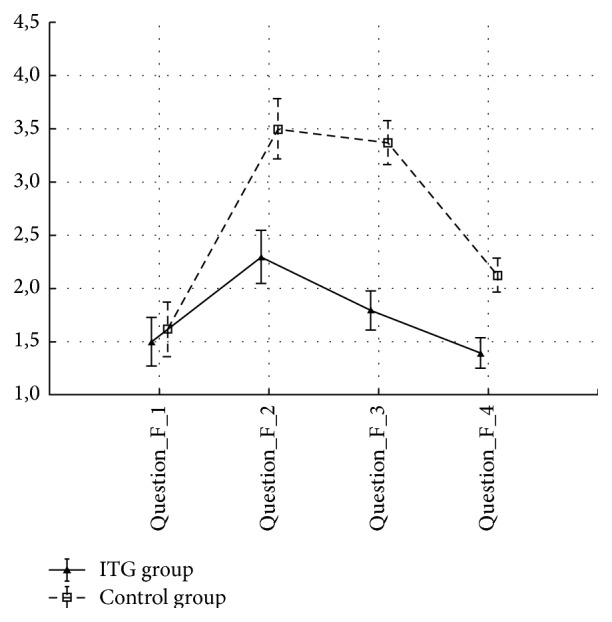
Means and SEM for question F: Do you have instability with, or does shopping in a supermarket bother you (rapid changes in products on the shelves)? Question_F_1: 3 months before surgery, Question_F_2: 3 weeks after surgery, Question_F_3: 3 months after surgery, and Question_F_4: 1 year after surgery. ANOVA 2-way (time × group) interaction effect *p* value < 0.05.

**Figure 3:**
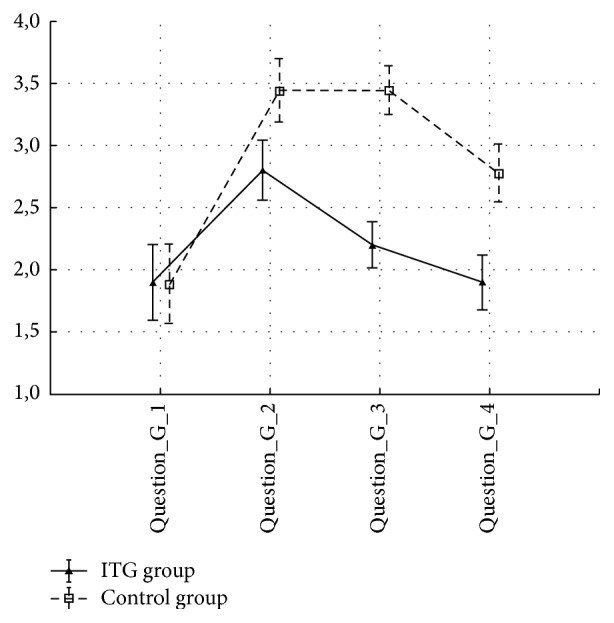
Means and SEM for question G: Do you have instability with, or does a greater amount of auditory and visual sensations bother you (e.g., shopping centers)? ANOVA 2-way (time × group) interaction effect *p* value < 0.05. Question_G_1: 3 months before surgery, Question_G_2: 3 weeks after surgery, Question_G_3: 3 months after surgery, and Question_G_4: 1 year after surgery. ANOVA 2-way (time × group) interaction effect *p* value < 0.05.

The gentamicin and control groups did not differ with regard to demographic and clinical variables (age, canal paresis, length of surgery, and size of tumor) prior to the surgery (all *p* > 0.05). All patients in the gentamicin group developed near vestibular loss (side difference in caloric test ranged from 80% to 92%) as evaluated by clinical and bithermal caloric testing. No patient had spontaneous nystagmus after gentamicin instillation; the majority reported instability one week after gentamicin application without nystagmus. There were no statistical differences between the control and gentamicin groups with regard to GBI, GHSI, and DHI results (Table 2). The DHI score was significantly worse in the gentamicin group in time 2 (*p* < 0.05) using the ANOVA test (Figure 1). There was statistical significant difference (*p*  value^b^ = 0.014) in subscale GBI social support but in subscale GBI general statistical significant difference was not confirmed. An important finding is that gentamicin-pretreated patients were significantly more resilient to a high sensorial load in shopping malls, as documented by low scoring on questions (F) and (G) (statistically significant *p* < 0.05, Figures 2 and 3).

## 6. Discussion

Vestibular rehabilitation consists of specific exercises which improve the recovery of vestibular function [6]. The idea of presurgical ablation of eventual residual vestibular function on the side of the tumor paralleled with rehabilitation [7] leads to separation of the two traumas of resection of vestibular schwannoma (e.g., acute vestibular loss and surgical trauma to cerebellum and brainstem).

If these two traumas run in parallel, there is risk of slower vestibular compensation. As such their separation should lead to a more efficient adaptation to vestibular loss [10]. The idea of chemical prehabituation is to achieve compensation before surgery. Based on these findings, we hypothesized that the total score of the GBI, GHSI, DHI, and an additional nine extra questions would differ between patients who did and did not receive gentamicin.

We did not find a statistically significant difference in the total score in these questionnaires; however, the total DHI score at time 2 was worse (*p* < 0.05) in the gentamicin group.

Results of DHI questionnaire clearly document the effectivity of the gentamicin pretreatment. There is increase of DHI score from 16.6 to 32.4 after the ITG treatment. This score is not further influenced by the surgery and remains stable at 31.4. In the control group the DHI score is not changed from time 1 to time 2 (no intervention) and the score doubles after surgery, with change from 21.4 to 43.0. These results can be explained as an onset of the vestibular failure following the ITG—before the surgical neurectomy. After the operation there is only minor deterioration in the pretreatment group, which is contrasted by severe symptoms of acute vestibular loss in the control group. In the long term these differences tend to equalize, however. Three months after surgery, DHI score in the untreated control group is higher, but the difference is not statistically significant.

Vestibular compensation is complex, multifaceted process, which includes adaptation to the sudden decline of labyrinth activity, substitution of the missing perception by other senses, changes in the postural strategy, and behavioral adaptations. Anxiety, depression, and social factors are further factors important for the final level of handicap after sudden vestibular loss including labyrinth surgery [11, 12].

In our study the social support postoperatively was perceived as significantly greater in the control group postoperatively. We can speculate that this difference could be due to the more intensive postsurgical handicap with need for higher support from family and caregivers. This point seems worth to be further elucidated during future follow-up visits. Nevertheless this difference does not translate in the overall handicap and functional restoration as seen in the other scores examined. On the contrary DHI score in the control group postoperatively shows tendency towards higher handicap, even if perceived social support is higher.

It is necessary to point out differences that differentiate our study from that of Magnusson et al. [7] in which patients underwent a home vestibular exercise program three times daily for two weeks, followed by intratympanic gentamicin application, and continuation of an intensive vestibular exercise program for 6–8 weeks. After confirmation of the vestibular deafferentation by vestibular testing, patients underwent surgery. It is known that the vestibular compensation process is activated simultaneously by progressive loss of vestibular function after administration of gentamicin and that motivation and physiotherapist supervision are very important to receive a good effect of vestibular rehabilitation. In our study, patients in both groups practiced vestibular home exercises according to a booklet and without supervision two months preoperatively; vestibular function was reevaluated by the clinical head impulse test and bithermal caloric test. This means that we were sure that the reduction of vestibular function was minimally 70%, which is different than in the Magnusson study where all patients had 100% side difference. Our maximum side differences were 92% in patients who received more than eight injections of gentamicin (>320 mg cumulative dose), which was stressful for the patient. This fact can be the reason for the different results between our and Magnusson's study. In our experience, it was practically impossible to achieve a 100% side difference. According to Mruzek et al. [13], vestibular compensation is achieved with adequate implementation of daily living activities and not by specific vestibular rehabilitation. According to these authors, vestibular rehabilitation does not affect the process of compensation.

Gentamicin-pretreated patients were significantly more resilient to a combined sensorial load when exposed to noise and optic flow stimulation in shopping aisles and crowds. Optic flow is a form of three-dimensional optokinetic stimulation with an important influence on balance and movement perception. It was shown that habituation and adaptation to changes and perturbations of optic flow are lower in elderly people and patients with balance problems [14, 15]. This finding is of potential functional importance in everyday life and for quality of life.

We can only speculate on the mechanisms of this finding, but knowing that there were no differences in peripheral vestibular deficit and the results of other questionnaires, it seems feasible to assume that changes in higher central processing of balance and vestibular function are responsible for the observed effect. It is probable that gentamicin pretreatment changes the patient's expectations and lowers level of anxiety in the postoperative period [16].

Another possible explanation could be that in pretreated ITG group lower visual dependency develops during the compensation period of vestibular loss. The reason for such evolution could be slow development of vestibular deficit in several steps, which increases the weight of vestibular afferents from contralateral labyrinth and makes high dependency on the visual inputs unnecessary [12, 17].

## 7. Conclusion

Vestibular prehabituation with preoperative gentamicin ablation of vestibular function does not significantly improve quality of life from the view of stability. However, since patients in the gentamicin group were significantly less sensitive to visual perception, results of this study left open some questions, which should be explored by future research: assessment of degree of visual dependency following surgery, detailed study of optokinetic response and smooth pursuit movements, and assessment of social status and its influence on the final compensation of vestibular deficit.

## Figures and Tables

**Table 1:** 

	Patients	Age (years)	Side (L: left, R: right)	Size of tumor	Canal paresis (%)	Length of surgery (hours)
ITG group	1	33	L	1	40	7
	2	47	L	4	41	6
	3	65	R	3	15	7
	4	59	R	2	30	7
	5	42	R	3	40	6
	6	40	L	4	28	8
	7	51	R	2	27	8
	8	56	L	3	34	6
	9	59	L	4	18	7
	10	52	L	2	20	8

Control group	1	43	L	3	9	7
	2	47	L	4	7	6
	3	60	R	4	15	9
	4	60	L	4	54	11
	5	54	L	3	40	6
	6	56	R	3	24	6
	7	44	L	2	23	8
	8	42	R	4	18	8
	9	52	L	4	40	9
	10	45	L	2	35	5

ITG: intratympanic gentamicin.

**Table 2:** Results.

		ITG group		Control group		
		Mean (SD)	*p* value^w^	Mean (SD)	*p* value^w^	*p* value^b^
Age	—	50.40 (9.94)	—	50.30 (6.98)	—	0.980

GHSI total	Time 1	59.81 (14.66)	*p* ^1,2^ = 0.099	61.70 (11.56)	**p** ^1,2^ = 0.005	0.753
	Time 2	54.67 (14.74)	*p* ^2,3^ = 0.842	51.06 (12.44)	*p* ^2,3^ = 0.665	0.574
	Time 3	55.28 (11.05)	*p* ^3,1^ = 0.144	49.54 (10.84)	**p** ^3,1^ = 0.002	0.257

GHSI general	Time 1	62.88 (18.75)	**p** ^1,2^ = 0.044	65.21 (12.62)	**p** ^1,2^ = 0.003	0.748
	Time 2	54.08 (18.85)	*p* ^2,3^ = 0.822	49.69 (14.69)	*p* ^2,3^ = 0.748	0.582
	Time 3	55.04 (11.91)	*p* ^3,1^ = 0.071	47.68 (14.51)	**p** ^3,1^ = 0.001	0.231

GHSI social support	Time 1	60.00 (8.61)	*p* ^1,2^ = 0.433	61.67 (7.03)	*p* ^1,2^ = 0.103	0.641
	Time 2	61.67 (8.96)	*p* ^2,3^ = 0.242	65.74 (8.78)	**p** ^2,3^ = 0.006	0.332
	Time 3	59.17 (9.17)	*p* ^3,1^ = 0.694	59.17 (7.30)	*p* ^3,1^ = 0.218	1.000

GHSI physical	Time 1	48.33 (24.78)	*p* ^1,2^ = 0.612	48.33 (21.44)	*p* ^1,2^ = 0.375	1.000
	Time 2	50.83 (22.38)	*p* ^2,3^ = 0.735	41.67 (20.83)	*p* ^2,3^ = 0.477	0.370
	Time 3	52.50 (21.89)	*p* ^3,1^ = 0.400	46.67 (16.29)	*p* ^3,1^ = 0.858	0.508

DHI	Time 1	16.60 (20.13)	**p** ^1,2^ = 0.015	21.40 (28.54)	*p* ^1,2^ = 0.822	0.669
	Time 2	32.40 (21.52)	*p* ^2,3^ = 0.872	20.00 (18.50)	**p** ^2,3^ = 0.001	0.184
	Time 3	31.40 (19.46)	**p** ^3,1^ = 0.022	43.00 (28.76)	**p** ^3,1^ = 0.001	0.305

GBI total	Time 2	−0.31 (5.28)	*p* ^2,3^ = 0.242	—	—	—
	Time 3	−0.97 (12.76)	—	−8.78 (18.79)	—	0.291

GBI general	Time 2	−2.78 (7.51)	*p* ^2,3^ = 0.242	—	—	—
	Time 3	−3.17 (18.49)	—	−20.83 (26.28)	—	0.099

GBI social support	Time 2	14.81 (21.15)	**p** ^2,3^ = 0.001	—	—	—
	Time 3	15.00 (19.95)	—	36.67 (15.32)	—	**0.014**

GBI physical	Time 2	−5.56 (14.43)	**p** ^2,3^ = 0.001	—	—	—
	Time 3	−8.33 (18.00)	—	−0.83 (33.90)	—	0.544

*Note*. SD: standard deviation; *p*  value^w^: within-group differences tested by LSD post hoc tests after repeated measures ANOVA; *p* ^1,2^: *p* value of the difference between time 1 and time 2; *p* ^2,3^: *p* value of the difference between time 2 and time 3; *p* ^3,1^: *p* value of the difference between time 1 and time 3; *p*  value^b^: between-group differences tested by independent-group *t*-test; ITG: intratympanic gentamicin.
